# Characterization and Selection of *Lactobacillus plantarum* and *Lactobacillus paracasei* for prevention of oral bacterial infections from Chinese pickle

**DOI:** 10.1186/s13568-021-01245-1

**Published:** 2021-06-09

**Authors:** Guochao Jia, Xiaofeng Liu, Aimin Zhi, Jingjing Li, Yuanfeng Wu, Yao Zhang

**Affiliations:** 1 School of Biological and Chemical Engineering, Zhejiang University of Science and Technology, 318 Liuhe Road, Hangzhou, 310023 Zhejiang China; 2 School of Chemical Engineering and Food Science, Zhengzhou University of Technology, Zhengzhou, 450044 Henan China; 3 College of Food Science and Technology, Zhejiang University of Technology, Hangzhou, 310014 Zhejiang China

**Keywords:** Lactobacillus, Oral health, Antibacterial activity, Probiotic properties, Fermented skim milk

## Abstract

The oral infections were mainly caused by *Streptococci* and *Staphylococcus aureus.* Antibiotic therapies can eliminate these harmful bacteria. However, it can break beneficial microbes and lead to the persistence of resistant strains. The objective of our study was to select potential probiotic strains for the prevention of oral bacterial infections and evaluate their potential probiotic properties in oral cavity. AR113 (*Lactobacillus plantarum*) and AR340 (*Lactobacillus paracasei*) with significantly antimicrobial *β-hemolytic streptococci* and *Staphylococcus aureus* activity were isolated from Chinese pickle through agar well diffusion assay. Through the analyses of probiotic properties in antibiofilm, lysozyme and hydrogen peroxide tolerance, bacterial surface properties, adherence ability, tooth degradation and anti-inflammatory activity, the AR113 and AR340 showed anti-adhesion activity of 45.2–71.1% and 20.3–56.8% against *β-hemolytic streptococci* and 15.4–52.6% and 30.7–65.9% against *Staphylococcus aureus*, respectively, at different concentration. The two strains with high hydrophobicity, autoaggregation and survival rate adhered strongly to FaDu cells. AR113 and AR340 exhibited low calcium released from teeth (0.04 μg/mL and 0.03 μg/mL, respectively). ELISA analysis showed that AR113 and AR340 significantly inhibited the LPS-induced increase of NO and TNF-α expression. Strains-fermented skim milk inhibited the growth of *β-hemolytic streptococci* or *Staphylococcus aureus*. AR113 and AR340 were considered as probiotic candidates because of their higher antibacterial activity against some oral pathogenic bacteria, no potential of primitive cariogenicity. These candidates were expected as new probiotics with potential oral health benefits and no harmful effects.

## Introduction

Oral health is an important element of general health and well-being. Although largely preventable, many people across the world still suffer unnecessarily from the pain and discomfort associated with oral diseases (Emfietzoglou et al. 2020). Oral infections constitute some of the most common forms of infections in humans (Mauramo et al. 2019). The infections of streptococcal and staphylococcal have emerged as a major source of morbidity and mortality (Peters et al. 2017). A number of distinct oral infections (e.g., angular cheilitis, parotitis and staphylococcal mucositis) are caused by these microorganism (Mccormack et al. 2015). Their impact is exacerbated by the epidemic-like emergence of resistant strains. In addition, antibiotic therapies can eliminate beneficial microbes and lead to the persistence of resistant strains (Hwang et al. 2017). New options for preventing and controlling oral infections caused by oral pathogens are urgently required.

The concept of microbial ecological change as a mechanism for preventing oral infections is important. Probiotic approach to eliminate oral pathogen is an alternative and promising way to combat infections by using harmless bacteria to displace pathogenic microorganisms. Probiotics play a pivotal role in normal body function and host health maintenance (Setbo et al. 2019; Mckenney and Pamer 2015). They alleviate the severity and duration of symptoms and reduce the incidence of oral infections (Franz et al. 2015; Kepert et al. 2016). It is shown that probiotics exhibited protective effect against various oral disorders. Probiotics can inhibit the number of *Streptococcus pyogenes* (Miettinen et al. 2008; Di et al. 2014), *Escherichia coli* (Polewski et al. 2016), *Porphyromonas gingivalis* (*P. gingivalis*) (Terai et al. 2015) and other microbial pathogens and decrease the risk of oral infections. In addition, many other beneficiary effects of administering probiotics in oral diseases have also been characterized, which includes maintenance of oral ecological balance (Jia et al. 2018), anti-inflammatory effects and immunomodulatory functions (Zupancic et al. 2017; Schmitter et al. 2018).

The probiotics play an important role in anti-oral diseases. However, evaluation criteria of probiotic properties and the mechanism in oral cavity are unresolved and needed to further research. The research described herein is part of a larger study to develop probiotics for oral health on a rational basis. The overall aim of this study is to select potential probiotic strains for the prevention of oral bacterial infections and evaluate their potential oral health benefits in vitro.

## Materials and methods

### Bacterial strains and culture conditions

*Lactobacillus plantarum* (*L. plantarum*) AR113 and *Lactobacillus paracasei* (*L. paracasei*) AR340 were isolated from Chinese pickle and were deposited at the China General Microbiological Culture Collection Center with preservation number CGMCC No. 13909 and CGMCC No. 15762, respectively. *β-hemolytic streptococci* CICC 10,373 and *Streptococcus mutans* (*S. mutans*) CICC 10,387 were obtained from the China Center of Industrial Culture Collection. *Staphylococcus aureus* (*S. aureus*) ATCC 29,213 was donated by Tongji University. Lactobacillus (LAB) strains were cultured in de Man, Rogosa and Sharpe (MRS) broth at 37 °C for 24 h. *β-hemolytic streptococci* strains were cultured on Columbia agar base plates supplemented with 5% sheep blood for 18 h at 37 °C under aerophilic conditions. *S. aureus* strains were plated onto Brian Heart Infusion (BHI) agar (Difco Laboratories, Detroit, MI, USA) for 24 h at 37 ℃.

### Isolation, screening and identification of LAB

In this experiment, samples were obtained from Chinese pickle. The liquid sample (1 mL) was suspended in saline blended vigorously and centrifuged at 3000*g* to settle the particulate matter. Appropriate decimal dilutions were prepared and poured into sterile Petri dishes on MRS agar. The media were incubated under anaerobic conditions for 24–48 h at 37 °C (Hwanhlem et al. 2014). Colonies with different morphologies on the MRS agar plate were selected and further subcultured in order to obtain a pure colony. Glycerol stock of LAB isolates were prepared and stored at – 80 °C. The preliminary identification of strains was made by Gram staining, cell morphology and catalase reaction (Angmo et al. 2016).

The LAB strains with antagonistic activity against oral pathogen *β-hemolytic streptococci* and *S. aureus* were examined by agar well diffusion methods (Arakawa 2019). Only strains showing maximum inhibitory activity against *β-hemolytic streptococci* and *S. aureus* were selected for identification to species level. 16S rRNA of selected strains was amplified by previous PCR procedure (Angmo et al. 2016). PCR primers 27F and 1492R were employed during amplification. The sequence of PCR product was carried out by the sequencing service of Sangon Biotech Co., Ltd (Shanghai). The 16S rRNA sequences were submitted to the GenBank nucleotide sequence database under accession No. MW750439 (AR113) and No. MW750442 (AR340). Sequence results were aligned with NCBI database using BLAST algorithm. Neighbor joining method was applied to determine the closest bacterial species using MEGA software 7.0.

### Antibiofilm assay

Antibiofilm properties of LAB strains against *β-hemolytic streptococci* and *S. aureus* were determined by previous method with minor modifications (Aarti et al. 2018). The culture was diluted 1:20 in medium. The 50 µL, 100 µL and 150 µL of suspensions were used to inoculate sterile 96 well polystyrene microtitre plates. After incubated for 24 h at 37 °C in 5% CO_2_, wells were washed with 0.1 M phosphate-buffered saline (PBS, pH 7.2) and stained with 1% crystal violet for 15 min. The wells were rinsed again, and the crystal violet was solubilized in 200 µL of ethanol-acetone (4:1, v/v). The absorbance was read at 620 nm using ELISA reader. The percentage reduction in the biofilm formation (B) by respective pathogens was calculated as: B = [(OD_b_ **–** OD_a)_ / OD_b_] × 100%, where OD_a_ is absorbance of well containing cell free neutralized supernatant and pathogens, OD_b_ is absorbance of well containing pathogens (Control).

### Lysozyme resistance

Lysozyme resistance to assess the in vitro ability of the strains to survive in the oral cavity was performed as previous described (Garcíaruiz, 2014). LAB strains were grown in MRS broth at 37 °C for 24 h. The cells were harvested by centrifugation, washed twice with PBS and resuspended in 2 mL of Ringer solution (8.5 g/L NaCl, 0.4 g/L KCl and 0.34 g/L CaCl_2_). To simulate the in vivo dilution by saliva, the bacterial suspensions (10^7^**–**10^8^ colonies forming units, CFU/mL)) were inoculated in a sterile electrolyte solution (0.22 g/L CaCl_2_, 6.2 g/L NaCl, 2.2 g/L KCl and 1.2 g/L NaHCO_3_) in the presence of 0.1 g/L of lysozyme. Bacterial suspensions without lysozyme were used as controls. Samples were incubated at 37 °C, and viable cell counts after 30 min and 120 min were enumerated on MRS agar by the drop plate method. Survival rates were calculated as a percentage of growth.

### Hydrogen peroxide resistance

The ability of the selected LAB strains to grow in presence of hydrogen peroxide was studied according to method of Kullisaar et al. with modifications (Kullisaar et al. 2002). Each strain was inoculated (2%, v/v) into 10 mL MRS broth containing 3% and 6% of hydrogen peroxide, all tubes were incubated at 37 °C. After 24 h of incubation, the residual viable population was calculated by plate counting on MRS agar. Survival rates were calculated as a percentage of growth.

### Bacterial surface properties

Autoaggregation capacities were performed using a previously described method (Collado et al. 2008). Hydrophobicity assays of the two selected strains were carried out using the method of Feng et al. (2017) with modifications. LAB were grown in MRS broth at 37 °C for 24 h and harvested by centrifugation (8000 g, 30 min). Cell suspension was collected and washed twice with NaCl solution (pH 7.0). Then cell suspension was adjusted OD_600nm_ to 0.2 by as preparation. An equal volume of dimethylbenzene was mixed into cell suspension. After mixed homogeneously for 120 s, the bacterial suspension was incubated at 37 °C for 30 min. Then, the mixture was again vortexed briefly and incubated at 37 °C for 1 h for phase separation. The aqueous phase was measured at the same wavelength. The percentage cell surface hydrophobicity (H) was calculated using the following equation: H = [(A_initial_ – A_final)_ / A_initial_] × 100%, where A_initial_ is the initial absorbance, A_final_ is the final absorbance.

### Adhesion to FaDu cell

The FaDu cell line was obtained from the Shanghai Institutes for Biological Sciences, Chinese Academy of Sciences (Shanghai, China). FaDu cell monolayers were grown in 3-cm petri plates on microscope cover glasses until they reached confluence. Prior to adherence assays, FaDu monolayers were washed three times with PBS. Subsequently, 1 mL lactobacilli suspension (10^7^ CFU/mL in RPMI-1640) and 1 mL antibiotic-free RPMI-1640 were added to each well and incubated at 37 ℃ in a 5% CO_2_ atmosphere. After 2 h of incubation, cells were washed three times with PBS, fixed with methanol, Gram-stained and then examined microscopically under oil immersion. The adherence index was evaluated in 20 random microscopic fields of adhering bacteria per 100 cells.

### Adherence activity to salivary-coated hydroxyapatite

The ability of the bacteria to adhere to salivary-coated hydroxyapatite (S-HA) was measured by a previously reported method with modification (Terai et al. 2015). Briefly, human saliva was filtered through a 0.22-μm filter (Merck Millipore) after being heated at 60 °C for 30 min and centrifuged (10,000*g*, 10 min, 4 °C), and S-HA beads were prepared by incubating HA beads in sterilized human saliva at 37 °C for 30 min with shaking. The FITC-labelled lactobacilli or *S. mutans* were re-suspended in PBS to adjust the OD_550nm_ to 1.0. Then 5 mg S-HA beads and 2 mL bacterial cell suspension were incubated at 37 °C for 60 min with shaking. After the test tube was left for 10 min for the S-HA beads to settle, 1 mL of the collected supernatant was mixed vigorously with 0.1 mL of a 0.1 M ethylene diamine tetraacetic acid solution to dissolve the remaining HA particles. Both the OD_550_ of the mixture and the control containing the bacterial cell suspension alone were measured. The adherence rate to the S-HA beads was calculated using the following formula: Adherence rate = [(OD_550nm *S. mutans*_ – OD_550nm lactobacilli_) / OD_550nm *S. mutans*_] × 100%. The adherence rate was used to determine whether the bacterium was adherent to S-HA.

### Tooth degradation

Tooth degradation assay of the selected LAB strains was conducted (Nikawa et al. 2004). 145 mg rat teeth sample were placed in 50 ml Centrifuge tube. Each well was inoculated with 300 μL of LAB suspension (6.0 × 10^8^ CFU/mL) or *S. mutans* suspension (3.0 × 10^9^ CFU/mL). Subsequently, 10 mL of BHI broth was carefully added and the resulting mixtures were incubated for 0, 3, 6, 12, 24 and 48 h at 37 °C. After incubation, the amount of calcium released was measured by using a commercial kit (CA590, Leadman, Peking, China) according the manufacturer’s instructions.

### Anti-inflammatory activity assay

Anti-inflammatory activity property of LAB were obtained by inhibition of nitric oxide (NO) and cytokines–tumor necrosis factor-α (TNF-α) production (Tellez et al. 2010). RAW 264.7 macrophage cells (Shanghai Institute of Life Science, Shanghai, China) were seeded on a 24-well plate at a concentration of 10^6^ cells/mL and incubated for 4 h at 37 °C in 5% CO_2_. Then, the culture media was mixed with fresh media (control) or media-containing LAB cells (10^4^ cells/mL). After 2 h of incubation, lipopolysaccharide (LPS) solution (final concentration 1 μg/mL) was added and incubated for 24 h. The levels of NO and TNF-α were measured by enzyme-linked immunosorbent assay (ELISA).

### Safety assessment and enzyme activity

Hemolysin production was detected using Columbia agar plates supplemented with 5% of sheep blood (Amersco, Solon, OH, USA). The presence of α or β-hemolysis was assessed by the formation of clear or greenish zones around the colonies, respectively.

Enzyme activity was measured using the commercially-available, semi-quantitative API-ZYM system (BioMérieux, Montreal, QC) as previously described. According to the manufacturer’s instructions, cell suspension was adjusted to McFarland standards 5. Then 65 μL of cell suspension were added into each well of the API-ZYM strip and were incubated at 37 °C for 4 h in anaerobic conditions. The results were graded based on the amount of from substrate hydrolyzed on a scale from 0 (no activity) to 40 (or ≥ 40 nM).

For antibiotic susceptibility testing, LAB strains (10^8^ CFU/mL) were inoculated onto MRS soft agar. Commercial antibiotic discs (amoxicillin, penicillin, tetracycline, erythromycin, gentamicin, clindamycin, and ofloxacin, provided by Sangon Biotech (Shanghai) Co., Ltd) were placed onto the agar and incubated at 37 °C for 24 h. Resistance or sensitivity was assessed according to the CLSI/NCCLS standard.

### Inhibition of Streptococci or S. aureus growth by fermented skim milk

Antimicrobial effects of the supernatant of fermented skim milk were tested against *β-hemolytic streptococci* or *S. aureus* using the agar well diffusion method (Arakawa 2019). Briefly, 100 μL of *β-hemolytic streptococci* or *S. aureus* culture (1 × 10^6^ cfu/mL) was plated onto the surface of a plate containing 20 mL of BHI agar. Then, 6-mm-diameter wells were uniformly bored in the BHI agar, and 100 μL of fermented skim milk supernatant (6000*g* for 15 min) was dispensed into each well. Plates were incubated at 37 °C for 24 h. After incubation, inhibition zone diameters surrounding each agar well were measured. Inhibition was considered positive when the zone diameter was > 6 mm.

### Statistical analyses

All experiments were conducted in triplicate. Data analysis was performed using SPSS statistical software (SPSS, Inc., Chicago, IL). All results were expressed as mean ± SD.

## Results

### Isolation, screening and identification of LAB

**Fig. 1:**
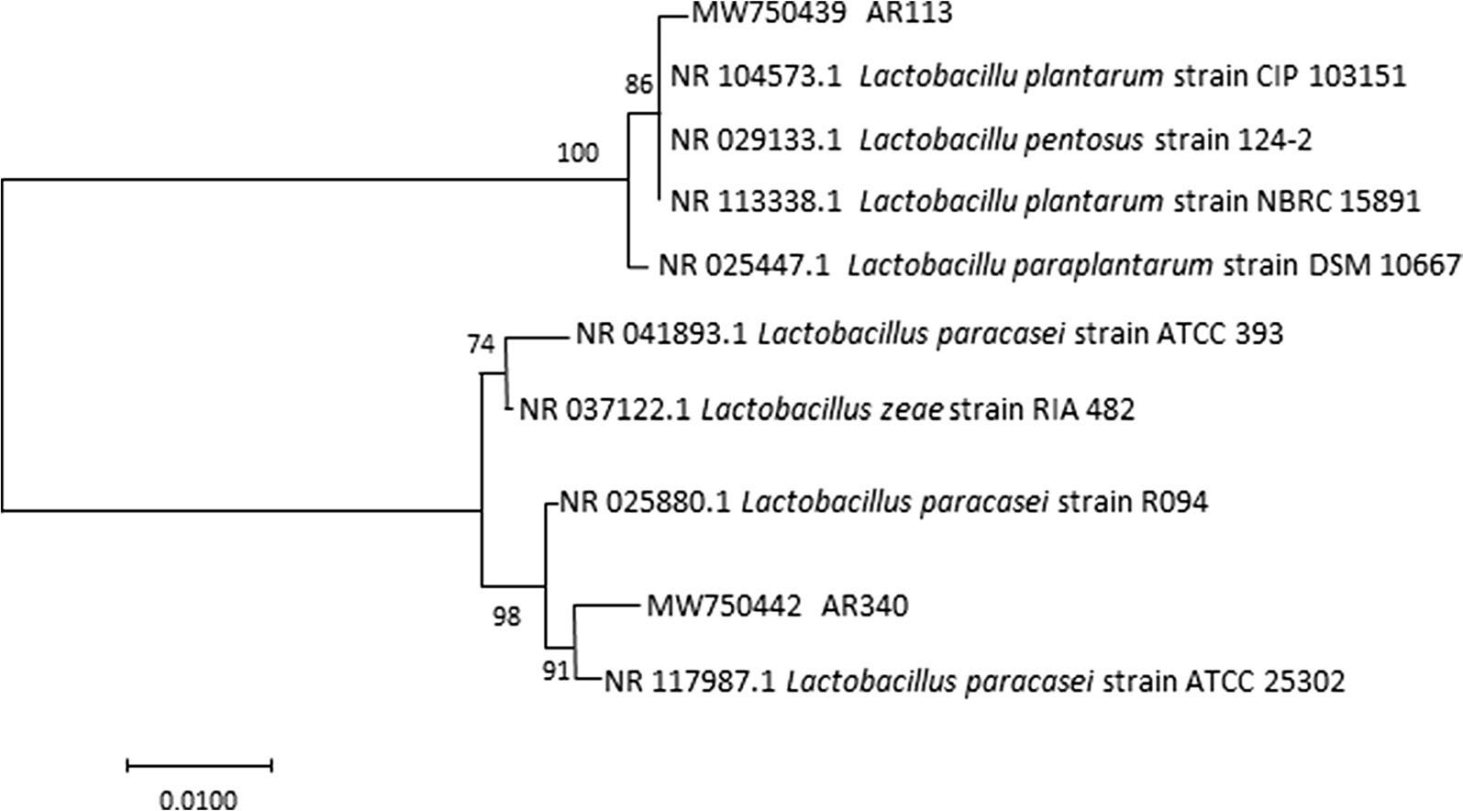
Neighbor joining phylogenetic tree based on 16S rRNA sequences

The 32 out of 81 isolates from Chinese pickle were identified to LAB based on their Gram reaction, morphology and catalase test (data not shown). All strains were recorded as catalase negative and Gram-positive. The morphology of the strains were cocci in pairs or long chains, and bacilli in pairs or chains. Of all the isolates, two strains showed the maximal antibacterial potential against *β-hemolytic streptococci* and *S. aureus*. They were identified to be *L. plantarum* (AR113) and *L. paracasei* (AR340), respectively (Fig. 1).

### Antibiofilm activity

**Fig. 2:**
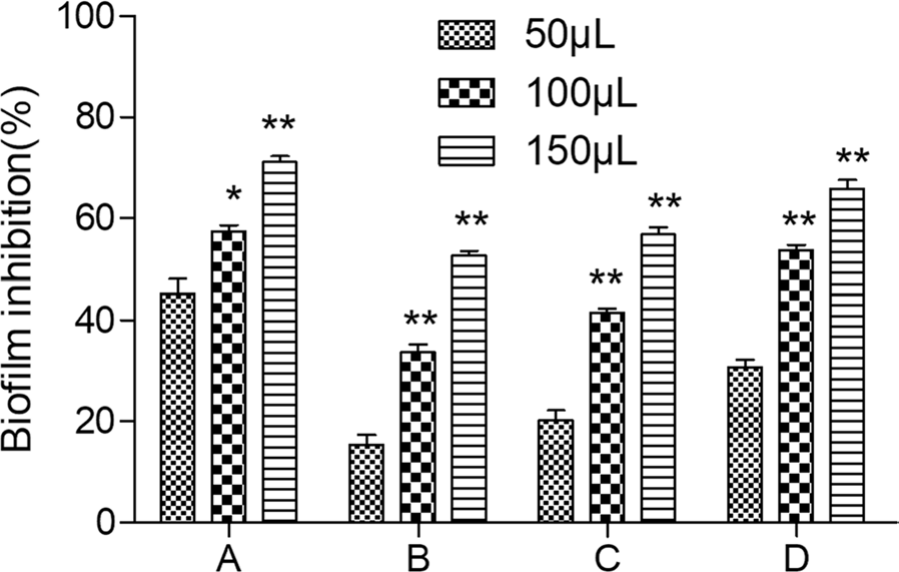
Antibiofilm activity of various concentrations of cell free neutralized supernatant of strains against pathogens. **A**, **B** represented the antibiofilm activity of AR113 strains against *β-hemolytic streptococci* and *S. aureus*. **C**, **D** represented antibiofilm activity of AR340 strains against *β-hemolytic streptococci* and *S. aureus*. Values are expressed as mean and SD (n = 3). * *p* < 0.05 and ** *p* < 0.01 compared with the biofilm inhibition of 50 μL cell free neutralized supernatant of strains against pathogens

The antibiofilm activities of selected strains against pathogens were shown in Fig. 2. Biofilm formations of pathogens were inhibited by the two strains at different concentration (50, 100 and 150 μL). The cell free neutralized supernatant of strain AR113 revealed higher anti-adhesion activity (45.2–71.1%) against *β-hemolytic streptococci* in a concentration dependent manner, followed by *S. aureus* (15.4–52.6%). However, AR340 revealed higher anti-adhesion activity (30.7–65.9%) against *S. aureus* in a concentration dependent manner, followed by *β-hemolytic streptococci* (20.3–56.8%).

### Resistance to lysozyme and hydrogen peroxide

**Fig. 3:**
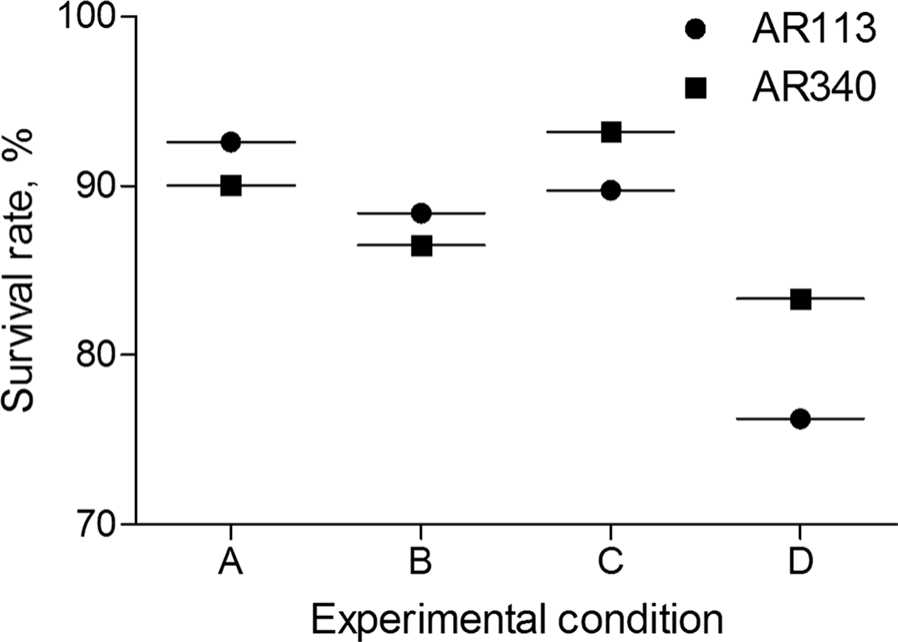
Lysozyme and hydrogen peroxide resistance of the selected strains. **A** A survival rate of strains after 30 min incubation with lysozyme. **B** A survival rate of strains after 120 min incubation with lysozyme. **C** A survival rate of strains in the condition of 3% hydrogen peroxide incubation. **D** A survival rate of strains in the condition of 6% hydrogen peroxide incubation

As shown in Fig. 3, survive rates of the strains in oral conditions were detected. The survival values ranged from 86.5 to 88.4% and from 76.2 to 83.4% of growth in the same medium with supplements for lysozyme and hydrogen peroxide, respectively. This result showed the high resistance of the two strains to 100 μg/mL of lysozyme under conditions stimulating the in vivo dilution by saliva and 6% of hydrogen peroxide hydrogen peroxide.

### Bacterial surface properties

**Fig. 4:**
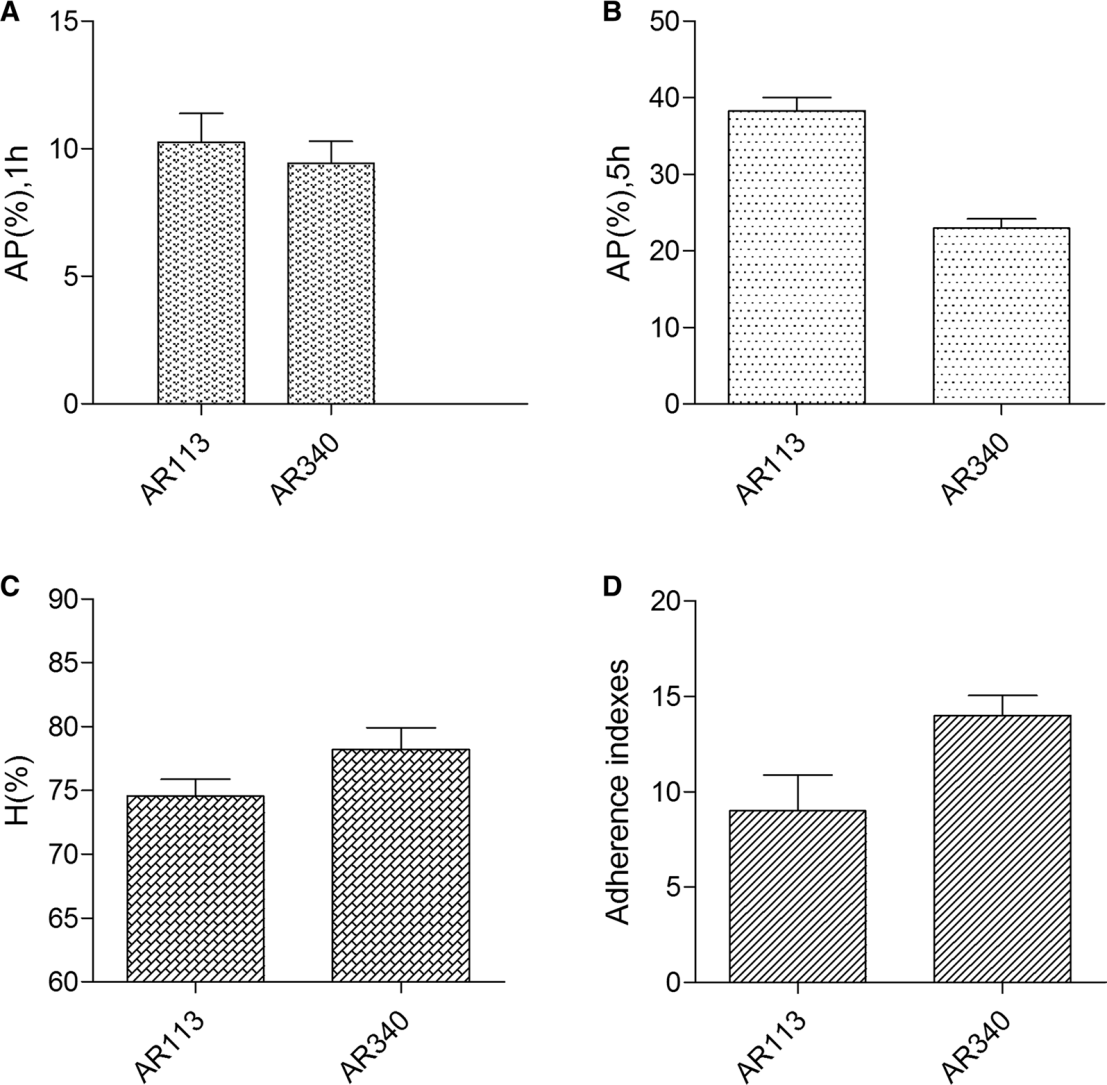
Bacterial surface properties and adhesion index of lactobacilli strains. **A** The autoaggregation of lactobacilli strains after 1 h of incubation. **B** The autoaggregation of lactobacilli strains after 5 h of incubation. **C** The hydrophobicity of lactobacilli was assessed. **D** The adhesion index of lactobacillus was assessed

Adhesion to epithelial cells is an important factor for the colonization of probiotic strains, because it provides a competitive advantage over other inhabitants. The percentage of cell surface hydrophobicity and auto-aggregation were presented in Fig. 4A–C. The two selected strains exhibited highly hydrophobic (> 74%). The percentages of LAB autoaggregation ranged from 9.4 to 10.3% and 22.9 to 38.3% after 1 h and 5 h of incubation, respectively. The related strains in the present study exhibited that autoaggregation abilities were enhanced with time and higher at 5 h of incubation than at 1 h.

### Adhesion to FaDu cell

Lactobacillus strains with antibacterial activity were further examined for the ability of adhering to FaDu cells. The adherence indexes of these strains were shown in Fig. 4D, AR113 and AR340 exhibited the high adherence capacity to FaDu cells. All the selected Lactobacillus strains showed substantially uniform distribution on the cellular surface with a certain degree of clusters or bacterial aggregates.

### Adherence to S-HA

**Fig. 5:**
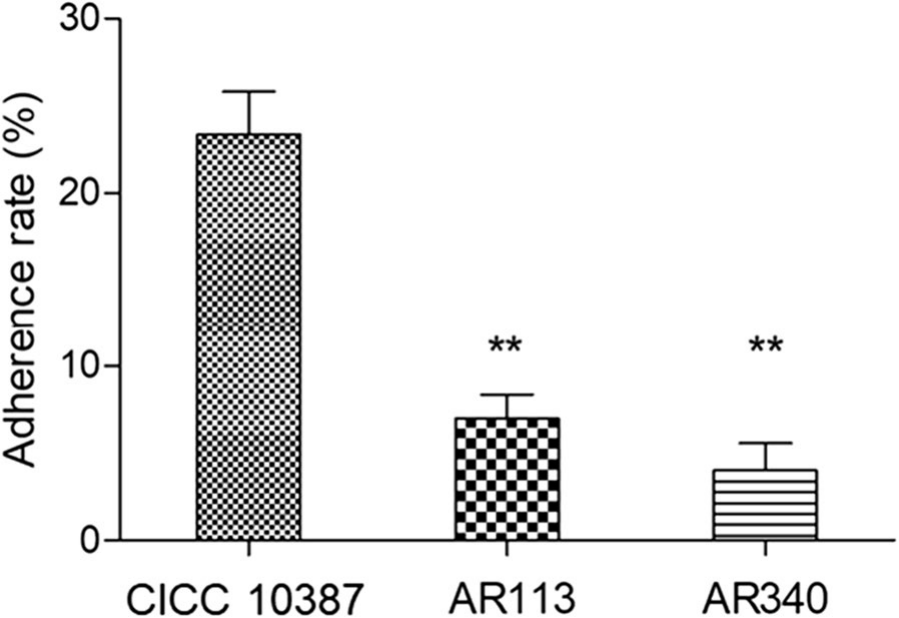
Adherence of LAB isolates to S-HA. Values are expressed as mean and SD (n = 3). * *p* < 0.05 and ** *p* < 0.01 compared with the adherence rate of *S. mutans* CICC 10387

The requisite for a microorganism to act as an oral probiotic was that it must be able to have a low adhesion rate to the tooth surface. S-HA was used to be oral model systems. Figure 5 showed the adherence of the LAB strains to S-HA. The adhesion rate of *S. mutans* CICC 10,387 to S-HA was 23.4%, and the adhesion rate of AR113 and AR340 to S-HA were relatively low, less than 7%.

### Calcium release from rat teeth

**Fig. 6:**
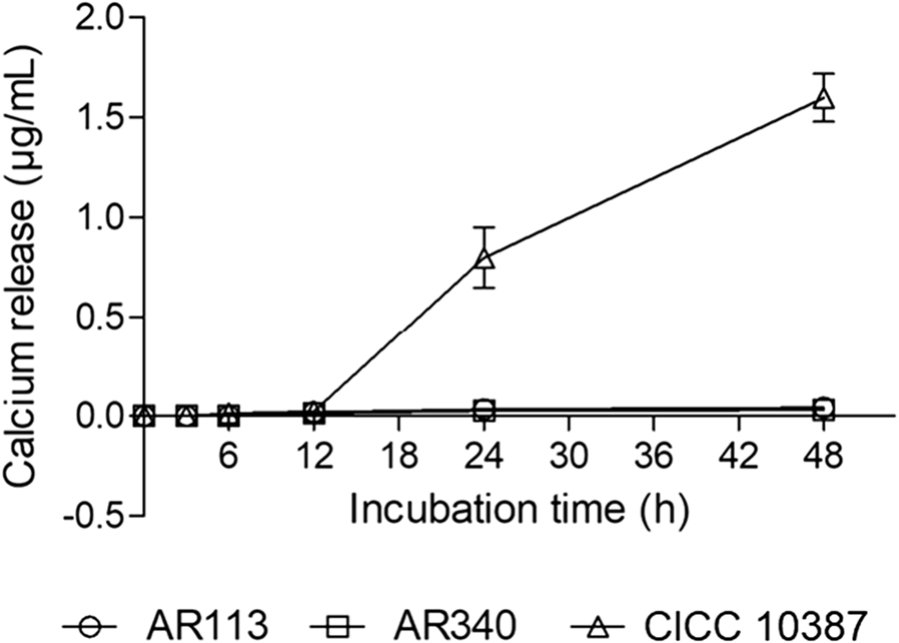
Effects on demineralization of rat tooth enamel by lactobacilli

We conducted a preliminary study to determine the degradation of teeth by lactobacilli (Fig. 6). The effect of *S. mutans* CICC 10,387 was negligible within 12 h of incubation, but the calcium release increased considerably. After 48 h of incubation, the concentration of calcium released from teeth caused by *S. mutans* CICC 10,387 (1.6 μg/mL). In contrast, the calcium release caused by AR113 and AR340 was negligible (0.04 μg/mL and 0.03 μg/mL, respectively) at 48 h of incubation.

### Anti-inflammatory activity assay

**Fig. 7:**
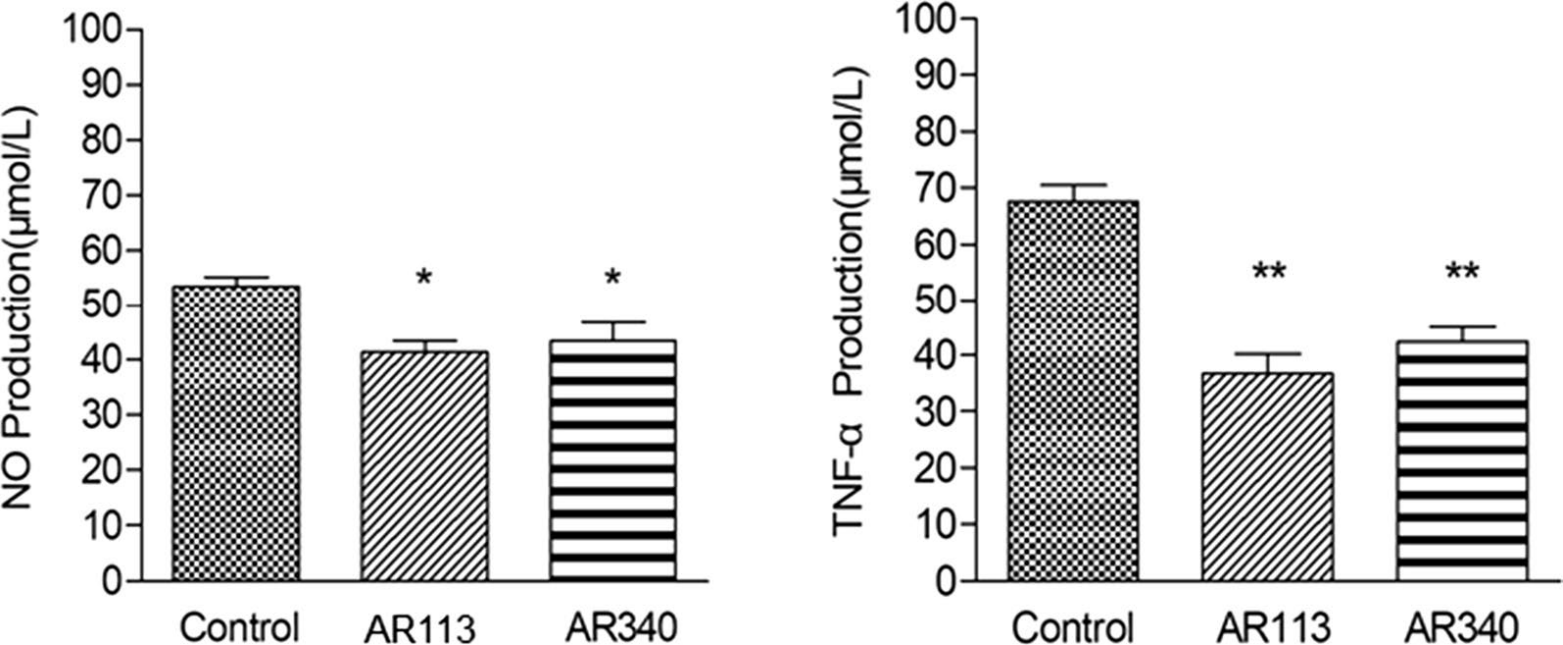
Effect of LAB strains on cytotoxicity and NO production of RAW 264.7 cells. NO and TNF-α production by RAW 264.7 cells treated with LPS, LPS + LAB strains Values are expressed as mean and SD (n = 3). * *p* < 0.05 and ** *p* < 0.01 compared with the control

We examined whether LAB could inhibit TNF-α and NO productions in RAW 264.7 macrophages. The endotoxin LPS is one of the most extensively studied inducers of the productions of NO and pro-inflammatory cytokines TNF-α in the macrophages. As shown in Fig. 7, macrophages cultured in the presence of LPS (1 μg/mL) generated 53.6 μmol/L NO and 67.5 μmol/L TNF-α. The selected strains exhibited anti-inflammatory activity. When compared with the controls, the amount of NO and TNF-α produced by macrophages co-cultured with LAB strains were reduced by approximately 19.0–22.8% and 37.2–45.6%, respectively. These results suggested that LPS-induced NO and TNF-α production was effective suppressed by the two LAB strains.

### Safety assessments

Trypsin, α-chymotrypsin and β-glucuronidase activity were not detected in any of the selected strains (data not shown). In addition, none of the four strains showed hemolytic activity. Since the transmission of antibiotic resistance genes to potentially pathogenic bacteria in the upper respiratory tract is of major health concern, we tested the selected LAB strains for sensitivity to low concentrations of commonly prescribed antibiotics. All of the selected LAB strains were susceptible to antibiotics that are routinely used for the control of oral infections, including amoxicillin, penicillin, tetracycline, erythromycin, gentamicin, clindamycin, and ofloxacin (data not shown). Thus, these four LAB strains can be applied to the oral cavity.

### The inhibition of AR113 and AR340-fermented skim milk

**Fig. 8:**
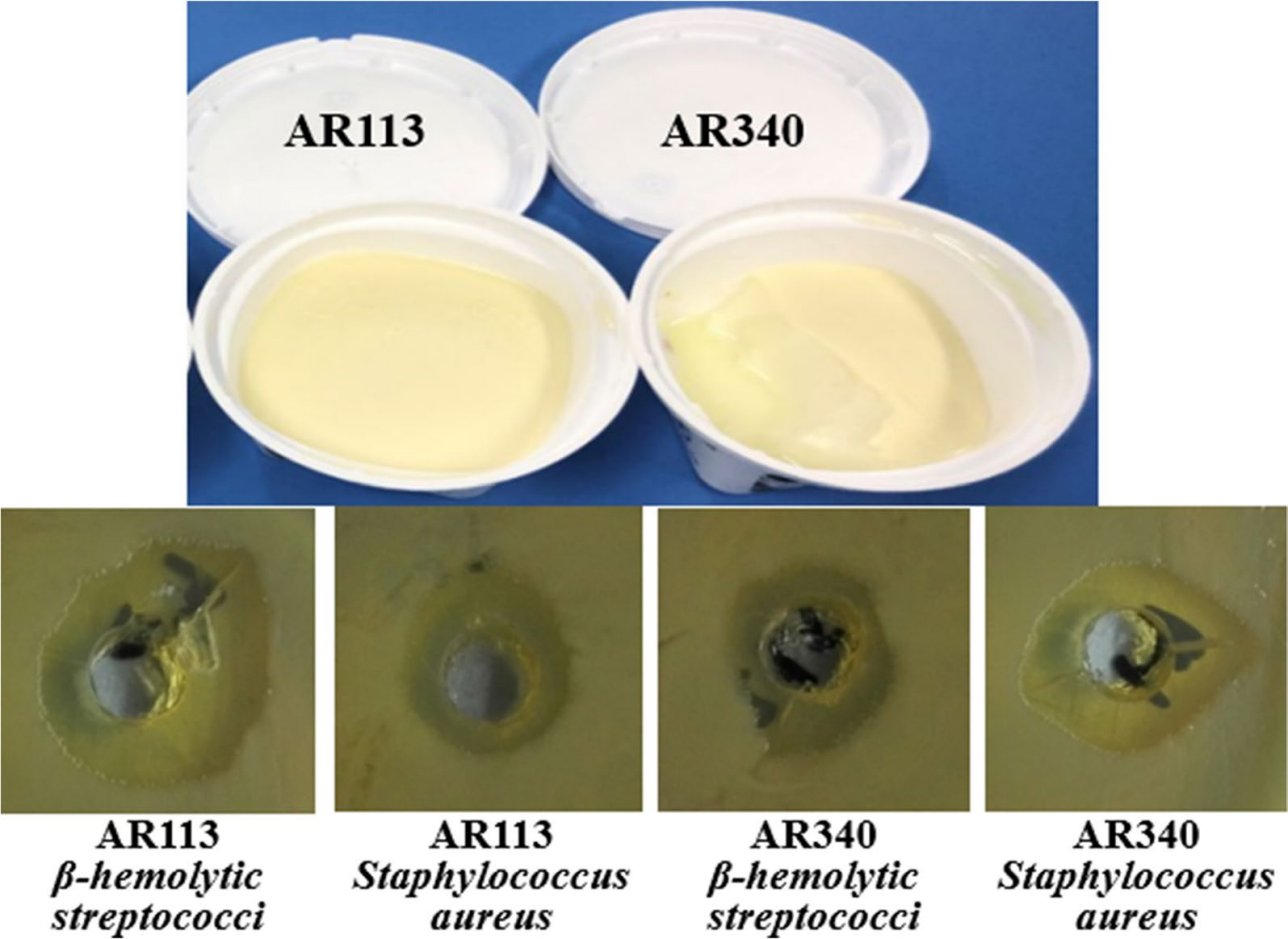
The inhibition of AR113 and AR340-fermented skim milk to *β-hemolytic streptococci* and *S. aureus*

AR113 and AR340-fermented skim milk inhibited the growth of *β-hemolytic streptococci *in vitro, with inhibition zone diameters ranging from 10.2 to 14.3 mm and from 9.3 to 11.0 mm, respectively. The inhibition zone diameters of AR113 and AR340-fermented skim milk to *S. aureus* ranged from 9.0 to 11.4 mm and from 10.6 to 13.1 mm, respectively (data not shown). It provided the basis for the development of new natural food antibacterial products using these LAB strains (Fig. 8).

## Discussion

Oral infections can be defined as infections occurring in different locations of the human oral cavity (Gendron et al. 2000). Microbial populations colonizing the oral cavity are a major source of pathogens responsible for oral infections. Each entity has distinct clinical and microbial features. The occurrence of oral bacterial infections has been increasing significantly in the last decades, contributing to high morbidity. Dental caries, periodontal diseases and streptococcal pharyngitis are the most common oral infectious diseases of man (Fani and Kohanteb 2017). Bacterial species associated with oral infections include *P. gingivalis*, *Prevotella intermedia*, *β-hemolytic streptococci* and *S. aureus*, et al. Given the problems associated with resistance to antibiotics has increased in recent years, the development of novel probiotic therapeutic strategies is critical for the prevention and treatment of oral bacterial infections (Llor and Bjerrum 2014). The aim of this study was to screen probiotic strains for the prevention of oral bacterial infections.

The 32 of 81 isolates from Chinese pickle were characterized as Lactobacillus genus. Among these LAB strains, only *L. plantarum* AR113 and *L. paracasei* AR340 proved to be effective in antagonizing oral pathogen *β-hemolytic streptococci* and *S. aureus*. They also showed significant anti-adhesion property against these pathogens*.* The biofilm formations of *β-hemolytic streptococci* and *S. aureus* were significantly inhibited by the supernatant of Lactobacillus. In general, the anti-adherence characteristics of pathogens by LAB are mainly due to the competition with the adhesion sites, and the effects bacteria growth inhibitory substances present in the supernatant of lactobacilli. Here, the anti-pathogen activity of strain might be due to the secretion of bioactive components from the isolate that inhibited the development of *β-hemolytic streptococci* and *S. aureus* (Fani and Kohanteb 2017).

The oral tolerance, bacterial adhesion, cariogenic potential and anti-inflammatory properties were tested to assess whether these lactobacilli have probiotic properties in oral cavity. Microorganisms to be applied as oral probiotic must overcome the inhospitable condition in oral cavity and subsequently colonize oral cavity. In order to reach active and viable enough through oral cavity, they should be resistant to lysozyme and hydrogen peroxide. The in vitro lysozyme and hydrogen peroxide tolerance study showed that the isolates showed resistance to 100 μg/mL lysozyme and 6% hydrogen peroxide and revealed that selected strains had the ability to survive in an artificial mouth system.

Considering the adhesion of lactobacillus strains to epithelial cells to be a crucially important factor for probiotics colonization and inhibition of pathogen adhesion (Angmo et al. 2016), the adhesion index of lactobacillus was assessed. AR113 and AR340 were found to adhere strongly to FaDu cells. Cell surface properties are indicative parameters for probiotic cells adhesion to epithelial cells (Abushelaibi et al. 2017). In this study, Lactobacillus strains were examined for degree of hydrophobicity and autoaggregation ability. The two strains exhibited some degree of autoaggregation and relatively high hydrophobicity index, which indicated that hydrophobic interaction might be involved in the adhesion of lactobacilli to oral epithelial cells. It was consistent with the previous studies that hydrophobicity and aggregation ability were correlated to cell adherence properties (Collado et al. 2008; Vidhyasagar and Jeevaratnam 2013; Angmo et al. 2016; Abushelaibi et al. 2017). It suggested that the AR113 and AR340 might protect the host epithelium by forming a barrier through self-aggregation and adherence mechanisms, by interfering with pathogen binding to host cell receptors, and by co-aggregation with potential pathogens (Kakisu et al. 2013; Tsai et al. 2017).

Tooth decay is still one of the most common oral diseases worldwide, although the proportion of the elderly population with many teeth is increasing in developed countries due to the development of daily dental care for the improvement of oral health (Terai et al. 2015). Tooth decay is initiated by the adherence of colonizers such as oral pathogenic bacteria *streptococcus sobrinus* and *S. mutans* to tooth surfaces to form a dental plaque. Therefore, oral probiotics must have no cariogenic potential, such as enamel demineralization associated with no or low adherence to S-HA or teeth. In our study, AR113 and AR340 showed low enamel demineralization and adherence to S-HA. Most of *Streptococcus saliva*, *Streptococcus parasanguinis* and *S. mutans* strains demonstrated adherence to S-HA, which was used as an alternative to human teeth. Adherence activity was lower in lactobacilli than in *streptococci*. Our observations were consistent with findings reported previously.

NO is a highly reactive free radical that is involved in several physical and pathological processes and plays an important role in the pathophysiology of various diseases (Tellez et al. 2010; Kakisu et al. 2013). The excessive production of NO often leads to many diseases physiological reactions. TNF-α, which is produced by activated macrophages and other cells, has a broad spectrum of biological actions on activities of target cells, both immune and nonimmune cells (Wang et al. 2004). Thus, TNF-α is considered a major inflammatory mediator with systemic inflammatory properties. In this study, supernatants of LPS-stimulated RAW 264.7 cells in the presence of LAB strains were examined for the production of NO and the proinflammatory cytokines TNF-α. There was no effect of LAB alone on the productions of TNF-α and NO in normal RAW 264.7 macrophages. However, when LAB cells were added to RAW 264.7 cells at 1 h before addition of 1 μg/mL of LPS, TNF-α and NO productions were inhibited. Therefore, we propose that the strains can be used as benefit strains to improve oral health due to their immunomodulatory and anti-inflammatory properties. This is in line with previous reports on the immune-promoting activity of *L. plantarum* strains (Wang et al. 2009; Kuda et al. 2009).

This study demonstrated that *L. plantarum* AR113 and *L. paracasei* AR340 were considered as probiotic candidates because of their higher antibacterial activity against some oral pathogenic bacteria, no potential of primitive cariogenicity. These candidates were expected as new probiotics with potential oral health benefits and no harmful effects. It can be widely used in various commercial food products such as fermented milk, fermented meat, cereal, fruit juice and ice cream. Further in vivo studies should be also performed to confirm its potential beneficial effects.

## Data Availability

The datasets used and/or analyzed during the current study are available from the corresponding author on reasonable request.

## Abbreviations

*P. gingivalis*: *Porphyromonas gingivalis*
*S. mutans*: *Streptococcus mutans*
*S. aureus*: *Staphylococcus aureus*
MRS: De Man: Rogosa and Sharpe
BHI: Brian Heart Infusion
PBS: Phosphate-buffered saline
S-HA: Salivary-coated hydroxyapatite
LPS: Lipopolysaccharide
ELISA: Enzyme-linked immunosorbent assay
*L. plantarum*: *Lactobacillus plantarum*
*L. paracasei*: *Lactobacillus paracasei*
